# Emergency Endoscopic Hemostasis Using the Endoscopic Mucosal Resection Technique for Severe Bleeding From Early Gastric Cancer: A Case Report

**DOI:** 10.7759/cureus.54429

**Published:** 2024-02-18

**Authors:** Kimitoshi Kubo, Xinhan Zhang, Ikko Tanaka, Noriko Kimura

**Affiliations:** 1 Department of Gastroenterology, National Hospital Organization Hakodate National Hospital, Hakodate, JPN; 2 Department of Pathology, National Hospital Organization Hakodate National Hospital, Hakodate, JPN

**Keywords:** gastric cancer, endoscopic mucosal resection, autoimmune gastritis, endoscopic hemostasis, bleeding

## Abstract

Bleeding from gastric cancer may lead to severe anemia and hypovolemic shock, and can be a life-threatening condition in affected patients; thus, achieving hemostasis is essential to improving their clinical course. While endoscopic hemostasis is recommended as the hemostatic modality of first choice, endoscopic hemostasis involving the endoscopic mucosal resection (EMR) technique is also being used, though under-reported. An 85-year-old man diagnosed with bleeding from gastric cancer was raced to our hospital for hemostasis. Emergency esophagogastroduodenoscopy (EGD) revealed a 45 mm-sized elevated lesion involving the coagula due to dripping bleeding from the surface of the posterior wall of the gastric lower body. EMR was performed without any technical difficulty, and hemostasis was achieved immediately. The patient was discharged without rebleeding. This case appears to support the usefulness of EMR as an emergency endoscopic hemostatic modality for severe bleeding from early gastric cancer.

## Introduction

Gastrointestinal malignancies are reported to account for upper gastrointestinal bleeding (UGIB) in 2-8% of affected patients [1]. Of these, gastric cancer accounts for 58% of malignancy-related UGIB cases, the majority of which are found to be at advanced stages and associated with higher mortality rates [2,3]. Given that bleeding from gastric cancer may lead to severe anemia and hypovolemic shock and can be a life-threatening condition in affected patients, achieving hemostasis is essential to improving their clinical course [4,5]. Of note, male gender, hypertension, chronic kidney disease, and current H. pylori infection are shown to be reliable predictors of bleeding in gastric cancer [6].

Endoscopic hemostasis involving thermal, mechanical, or injection therapy is the treatment choice for UGIB [5]. However, it is reported that rebleeding occurs in 30-40% of patients who underwent endoscopic hemostasis, with early rebleeding shown to be associated with poor prognosis in these patients [4,7,8]. Severe bleeding from early gastric cancer is rare and, to date, its endoscopic hemostasis using the endoscopic mucosal resection (EMR) technique has not been reported. We herein report a case in which emergency endoscopic hemostasis was performed using the EMR technique for severe bleeding from early gastric cancer.

## Case presentation

An 85-year-old man with a history of hypertension was raced to a nearby hospital for hematemesis. Found to be severely anemic (Hb, 4.7 g/dL), he was admitted to the hospital for a blood transfusion. Emergency esophagogastroduodenoscopy (EGD) revealed blood oozing from gastric lesions in the gastric angle but the procedure could not provide hemostasis. Tarry stool persisted, and despite the transfusion of six units of blood over the next two days, the patient’s Hb remained at 4.9 g/dL. With additional blood transfusion (four units) and fresh frozen plasma (FFP) (four units) administered, he was transferred to our hospital for hemostasis involving surgery. Still found severely anemic (Hb, 6.6 g/dL) at this point, he was admitted to the intensive care unit (ICU).

**Figure 1:**
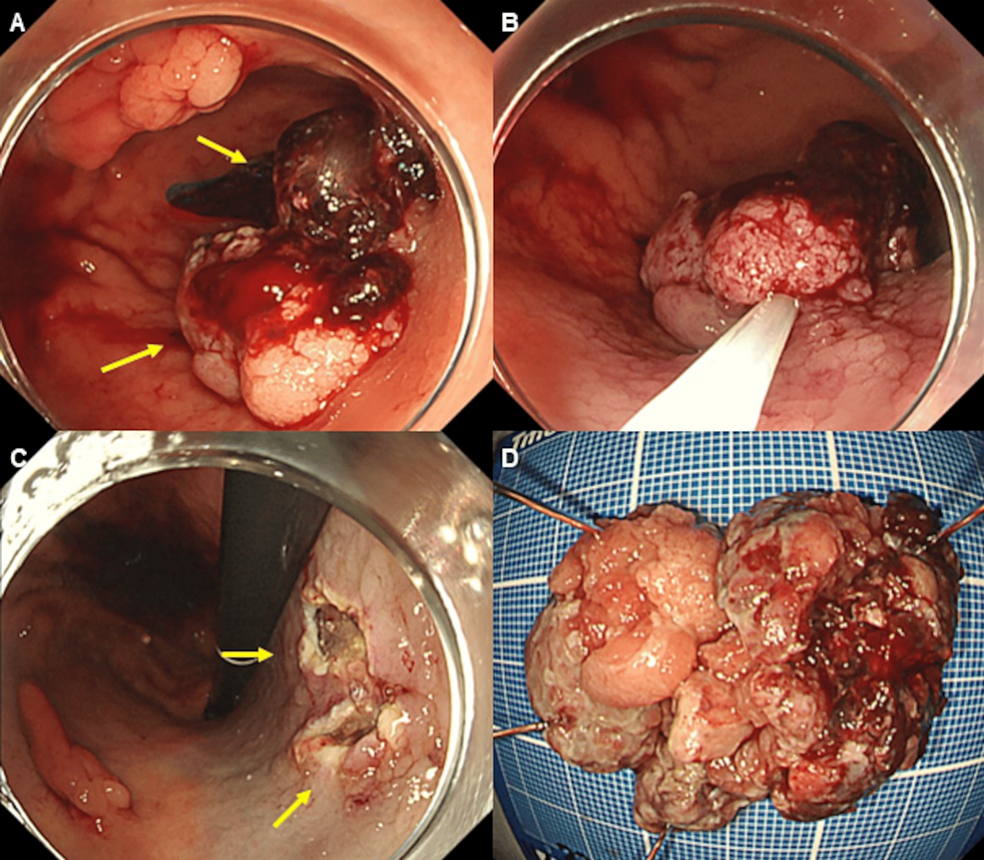
Esophagogastroduodenoscopy (EGD). Emergency EGD revealed a 45 mm-sized elevated lesion (arrows) with coagula attached due to blood dripping from the surface of the posterior wall of the gastric lower body (A). Endoscopic mucosal resection (EMR) was performed, and hemostasis was achieved immediately (B-D).

Emergency EGD revealed a 45 mm-sized elevated lesion involving coagula associated with blood dripping from the surface of the posterior wall of the gastric lower body, as well as a 15 mm-sized elevated lesion in the anterior wall of the gastric lower body, on white light imaging (WLI) (Figure 1A). EMR was performed, with an abdominal surgeon present, for endoscopic diagnosis of bleeding from early gastric cancer, as well as for hemostasis, which was achieved immediately (Figure 1B-1D).

**Figure 2:**
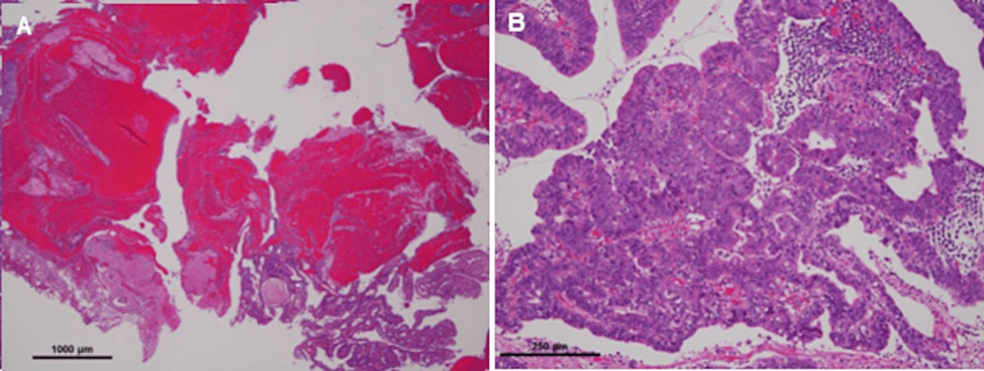
Pathological findings. Histological examination revealed the lesions to be a papillotubular adenocarcinoma (pap-tub), type 0-I, measuring 45 x 30 mm, pT1a (M) (3A, bleeding area; 3B, tumor area).

Histological examination of the lesion from the posterior wall of the gastric lower body showed a papillotubular adenocarcinoma (pap-tub), type 0-I, measuring 45 x 30 mm, pT1a (M) (Figure 2A, bleeding area; Figure 2B, tumor area). Histopathology of the second lesion obtained from the anterior wall of the gastric lower body was consistent with papillotubular adenocarcinoma (pap-tub), type 0-Ip, measuring 15 mm, pT1a (M).

**Figure 3:**
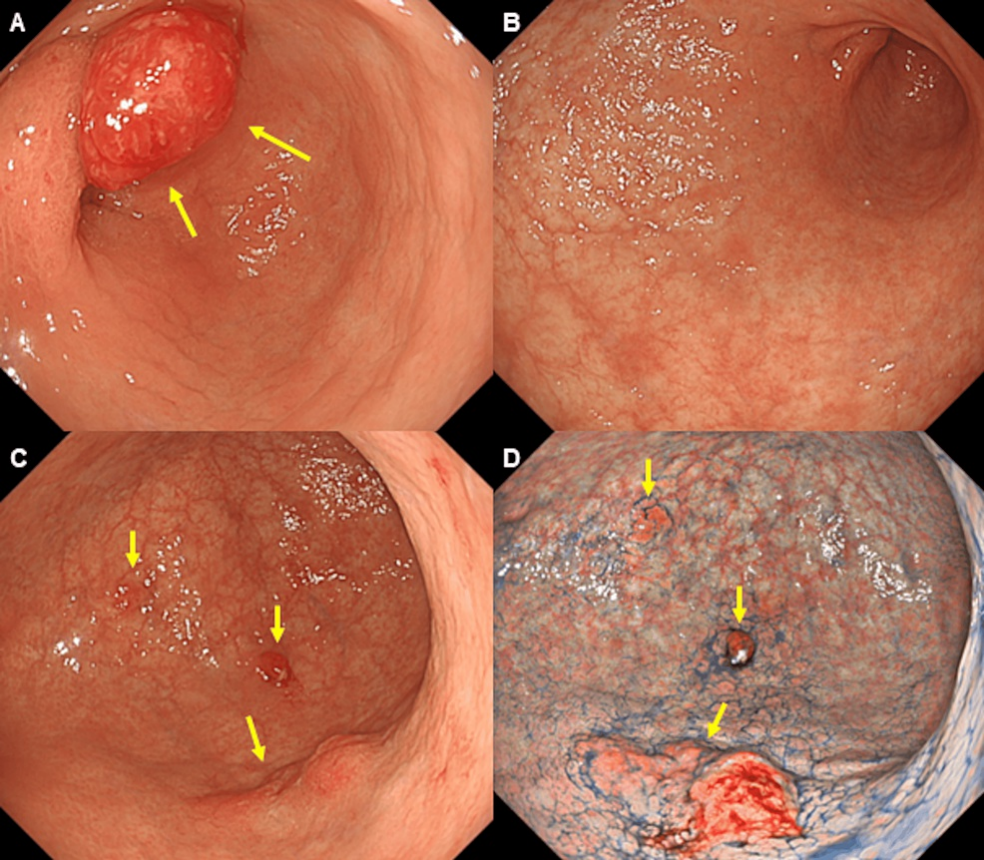
Repeat esophagogastroduodenoscopy (EGD). A repeat EGD performed 5 days after hemostasis revealed a 20 mm-sized, hyperplastic polyp in the anterior wall of the prepyloric lesion (3A), pan-atrophy without atrophic border (3B), small hyperplastic polyps in the greater curvature of the gastric angle (3C) and a 20 mm-sized, reddish, elevated lesion in the posterior wall of the gastric angle on WLI (3C), which was highlighted as a reddish, elevated and depressed type lesion (3D) on texture and color enhancement imaging mode 1 with indigo-carmine dye and biopsy confirmed as an adenocarcinoma.

A repeat EGD performed 5 days after hemostasis revealed a 20 mm-sized hyperplastic polyp in the anterior wall of the prepyloric lesion (Figure 3A), pan-atrophy without atrophic border (Figure 3B), small hyperplastic polyps in the greater curvature of the gastric angle (Figure 3C), and a 20 mm-sized, reddish, elevated lesion in the posterior wall of the gastric angle on WLI (Figure 3C). On texture and color enhancement imaging mode 1 with indigo-carmine dye, the lesion was highlighted as a reddish elevated and depressed type lesion (Figure 3D), which biopsy revealed as adenocarcinoma. He was shown to be positive for anti-parietal cell antibodies but negative for H. pylori on a stool antigen test and the biopsy, suggesting the presence of autoimmune gastritis. To accommodate the availability of his family living far away, the patient was discharged on day 19 of hospitalization without rebleeding and was readmitted 2 months later for surgical treatment of early gastric cancer and hyperplastic polyp. Endoscopic submucosal dissection (ESD) was performed on early gastric cancer in the posterior wall of the gastric angle, and EMR was performed on the hyperplastic polyp in the anterior wall of the prepyloric lesion. Histological examination revealed the first lesion obtained from the posterior wall of the gastric angle to be an adenocarcinoma, pap, type 0-IIa+IIc, measuring 20' 16 mm, pT1a (M), with no lymphovascular invasion. Histological examination of the second lesion obtained from the anterior wall of the prepyloric lesion showed a hyperplastic polyp.

The patient was thus diagnosed with synchronous triple gastric cancer associated with autoimmune gastritis. Since then, the patient has been visiting our hospital regularly for 3 years and taking a potassium-competitive acid blocker. A follow-up EGD and computed tomography examinations have shown no evidence of recurrence to date.

## Discussion

This case highlights offers two important facts. Firstly, hemostasis using EMR may represent an option in patients with bleeding from early gastric cancer. Over the past 10 years, there have been five studies on endoscopic hemostasis for bleeding from gastric cancer [4,7-10]. Injection therapy, mechanical therapy, thermal therapy, hemostatic powder, and combinations of these therapies have been reported as modalities for endoscopic hemostasis, with the success rates and rebleeding rates shown to range between 31% and 100% and between 18% and 41%, respectively [4,7-10]. In these studies, transcatheter arterial embolization or surgery was performed in patients failing endoscopic hemostasis. Song IJ. et al reported that transfusion of ≥ 5 units of red blood cells (RBCs) was a significant risk factor for rebleeding with the median overall survival after initial hemostasis shown to be lower in patients with rebleeding than in those without rebleeding (2.7 vs. 3.9 months, P = 0.020) [8]. In the present case, the patient was elderly and had already received 10 units of RBCs, and his risk of rebleeding with conventional endoscopic hemostasis was considered to be high. In addition, the gastric lesion was endoscopically diagnosed with early gastric cancer, and endoscopic hemostasis was achieved by resection using the EMR technique.

Secondly, in our patient, synchronous triple early gastric cancer had arisen from the atrophic mucosa associated with autoimmune gastritis, which is a rare occurrence. The present case was diagnosed as autoimmune gastritis which met the Japan Gastroenterological Endoscopy Society (JGES) diagnostic criteria: (A) endoscopic findings consistent with those for autoimmune gastritis (a predominance of severe mucosal atrophy extending from the gastric body to the fundus); and (B) gastric antibody positivity (e.g., anti-parietal cell antibodies) [11]. A recent study reported a pooled annual gastric cancer incidence of 0.14% in autoimmune gastritis, with its overall relative risk for gastric cancer being 11.05 [12]. In addition, Kitamura et al. characterized gastric cancers associated with autoimmune gastritis as protruded types, involving large tumor sizes, upper tumor locations, and papillary pathological types, with most of these being intramucosal [13]. The synchronous triple gastric cancers diagnosed in this case were shown to be consistent with the characteristics noted above.

## Conclusions

The endoscopic and pathological images offered here should provide a clear illustration of the case made for endoscopic hemostasis using the EMR technique. Further studies are needed to determine whether endoscopic hemostasis using the EMR method may prove useful for severe bleeding from early gastric cancer. In addition, early gastric cancer may present as a synchronous lesion associated with autoimmune gastritis. Clinicians need to be aware of the characteristics of early gastric cancer associated with autoimmune gastritis.
